# Prevalence and risk factors for *Plasmodium falciparum *malaria in pregnant women of eastern Sudan

**DOI:** 10.1186/1475-2875-4-18

**Published:** 2005-04-13

**Authors:** Ishag Adam, Amar H Khamis, Mustafa I Elbashir

**Affiliations:** 1 New Haifa Teaching Hospital, P. O. Box 61, New Haifa, Sudan; 2 Albayan College for Science, Sudan University for Science and Technology, Sudan; 3 Department of Biochemistry, Faculty of Medicine, University of Khartoum, Sudan

## Abstract

**Background:**

Pregnant women are more susceptible to malaria, which is associated with serious adverse effects on pregnancy. The presentation of malaria during pregnancy varies according to the level of transmission in the area. Our study aimed to demonstrate the prevalence and risk factors for malaria (age, parity and gestational age) among pregnant women of eastern Sudan, which is characterized by unstable malaria transmission.

**Methods:**

The prevalence and possible risk factors for *Plasmodium falciparum *malaria were investigated in 744 pregnant Sudanese women attending the antenatal clinic of New Haifa Teaching Hospital, eastern Sudan, during October 2003-April 2004.

**Results:**

A total 102 (13.7%) had *P. falciparum *malaria, 18(17.6%) of these were severe cases (jaundice and severe anaemia). Univariate and multivariate analysis showed that, age and parity were not associated with malaria. Women who attended the antenatal clinic in the third trimester were at highest risk for malaria (OR = 1.58, 95% CI = 1.02–2.4; P < 0.05).

Women with malaria had significantly lower mean haemoglobin (9.4 g/dl, 95% CI 9.1–9.7 versus 10.7, CI 10.6–10.8, P < 0.05). A significantly lower haemoglobin was observed in those with severe falciparum malaria compared to non-severe form (8.3 g/dl, 95% CI 7.6–9.1 versus 9.4, 95% CI 9.1–9.7, P = < 0.05).

**Conclusion:**

The results suggest that *P. falciparum *malaria is common in pregnant women attending antenatal care and that anaemia is an important complication. Preventive measures (chemoprophylaxis and insecticide-treated bednets) may be beneficial in this area for all women irrespective of age or parity.

## Background

Pregnant women are more susceptible to malaria, which causes serious adverse effects including abortion, low birth weight and maternal anaemia. It is the leading cause maternal mortality in Sudan [1-7].

The presentation of malaria during pregnancy varies according to the pre-existing immunity of the mother. Women living in areas of low transmission have little immunity to malaria which can cause severe syndromes, such as cerebral malaria and pulmonary oedema. In contrast, those who live in areas of stable malaria transmission enjoy greater immunity and experience fewer symptoms during episodes of malaria, although they commonly develop severe anaemia as consequence of the infection [1,2,5,8,9].

Understanding the epidemiology of malaria during pregnancy provides important insight into relevant immunological processes and facilitates decision on control strategies. Although there are extensive studies in highly endemic African countries [1,2,5,6,10-12], there is little published data available for Sudan, an African country where *P. falciparum *malaria transmission is unstable in the eastern region [13]. The study was conducted to investigate the prevalence and associated risk factors for *P. falciparum *malaria in pregnant women from eastern Sudan.

## Patients and methods

### Data collection

Pregnant women attending antenatal clinic (booking visit) of the New Haifa Teaching Hospital, eastern Sudan, during the period October 2003-May 2004 were approached to participate in the study. After verbal consent, questionnaires were administered requesting demographic informations on age, parity, gestational age and history of maternal illness. Gestational age was calculated from the last menstrual period and confirmed by ultrasound, when clinically indicated. Physical examination was completed to identify signs of severe malaria [14] and also obstetrical examination (blood pressure, pallor, fundal level and foetal heart sound).

### Laboratory methods

Thick and thin blood films were prepared from capillary blood, stained with Giemsa and 100 oil immersion fields were examined. Parasite density was determined by counting parasites and 200 leucocytes, assuming each woman has 6,000 leucocytes/μl. All the slides were double-checked blindly.

The haemoglobin concentration was estimated by the haematic acid method [15] in the first two months and, subsequently, using Haemocure haemoglobinometer (HemCue AB, Angelhom, Sweden). Ferrous sulfate (200 mg/day) and folic acid tablets (0.25 mg/day) were supplied.

### Ethical clearance

The study received ethical clearance from the Research Board of the Faculty of Medicine, University of Khartoum.

### Statistical analysis

Data were entered in a computer using SPSS for windows. Comparisons between means and percentages were done by Students' *t*-test, ANOVA, *X*^2 ^and Fisher's exact tests as appropriate. P < 0.05 was regarded as significant. Multivariate logistic regression was performed with malaria as the dependent variable, using age, parity and gestation as independent variables. These variables were categorized and used as follows: median age, ≤ 25 years versus >25 years); gravidae as primigravidae, secundigravidae, multigravidae (3–5)or grandmultigravidae >5; gestation as first (< 14 weeks), second (14–28 weeks) and third (> 28 weeks) trimester.

## Results

### Malaria and pregnancy

A total of 744 pregnant women attended the antenatal clinic of New Haifa Teaching Hospital during the study, 29.5% were primigravidae.

Table 1 summarizes patient characteristics for all sub-groups. 102 (13.7%) of women were infected with *P. falciparum*, 18(17.6%) of these were severe cases (jaundice and severe anaemia). Malaria prevalence and intensity (parasite count) were not significantly different amongst the different gravidity sub-groups (P > 0.05). The highest prevalence (18.3%) occurred in grandmultigravidae and the highest intensity (11,511 parasites/μl) was observed in primigravidae (Table 1).

**Table 1:** Characteristics of pregnant women in the study subjects *

Characteristics	Total	Primi-gravidae	Secundi-gravidae	Multi-gravidae	Grand-multigravidae	P Value
	
	N (744)	N (220)	N (131)	N (322)	N (71)	
Age (years)	26.6(9.9)	21.2(2.9)	25.7(2.3)	28.2(3.8)	34.9 (3.2)	< 0.001
Gestation (weeks)	27.7 (7.7)	27.3(8.09)	28.1(6.9)	27.6(7.6)	28.3(28.3)	0.18
Haemoglobin (g/dl)	10.5(1.4)	10.5(1.3)	10.3(1.6)	10.6(1.3)	10.3(1.2)	0.2
Malaria (total)	102(13.7)	32(14.5)	14(10.7)	43(13.4)	13(18.3)	0.4
Non severe	84(11.3)	28(12.7)	9(6.9)	34(10.6)	13 (18.3)	0.08
Severe	18(2.4)	4(1.8)	5(3.8)	9 (2.8)	0	0.3
Parasite count (parasites/μl)	6,586 (19,640.2)	11,511 (3,222)	6,817 (14,383)	3,504 (7,729)	4,497 (7,748)	0.4

*Data as mean (SD) or number (%) as appropriate

Mean (SD) age (26.2 ± 5.7 years versus 25.9 ± 5.3 years, P > 0.05) and parity (2.4 ± 2.4 versus 2.1 ± 2.1, P > 0.05) were not significantly different between infected and non-infected women. Two of 18 (11.1%), 29 of 290 (10%) and 71 of 436 (16.3%) women were infected in the first, second and third trimester respectively (P = 0.05).

### Factors associated with malaria infection

Univariate and multivariate analysis indicated that, age and parity were not significantly associated with malaria infection. The third trimester was significantly associated with malaria infection (OR = 1.58, 95% CI = 1.02–2.4; P < 0.05)(Table 2).

**Table 2:** Risk factor analysis

	Univariate			Multivariate		
**Variable**	OR	95% CI	P	OR	95% CI	P

**Age ≤ 25**	1.03	0.68–1.5	0.40	0.90	0.52–1.6	0.74
**Parity**						
Primigravidae	1.10	0.70–1.7	0.60	0.76	0.32–1.8	0.55
Secundigravidae	0.72	0.39–1.3	0.20	0.50	0.21–1.4	0.22
Multigravidae	0.96	0.62–1.4	0.80	0.74	0.37–1.4	0.41
Grandmultigravidae	1.40	0.77–2.7	0.20	3.20	0.27–46.2	0.30
**Gestational age**						
First trimester	0.61	0.27–1.3	0.15	0.51	0.22–1.1	0.11
Second Trimester	0.71	0.45–1.3	0.09	0.67	0.41–1.08	0.10
Third trimester	1.50	1.02–2.4	0.02	1.58	1.02–2.4	0.03

OR: odds ratio

### Malaria and haemoglobin

Mean haemoglobin was significantly lower in infected women (9.4 g/dl, 95% CI 9.1–9.7versus 10.7, CI 10.6–10.8, P < 0.05). A lower haemoglobin also occurred in women with severe malaria (9.4 g/dl, 95% CI 9.1–9.7versus 8.3, 95% CI 7.6–9.1 P < 0.05).

## Discussion

This study investigated the morbidity pattern of *P. falciparum *malaria during pregnancy in an area of New Haifa, which is characterized by unstable transmission [13]. The malaria prevalence was 13.7% and 18 women (17.6%) were severe cases. With the exception of the neighbouring country Ethiopia [6], malaria prevalence was much lower than in other African countries characterized by intense malaria transmission [10-12]. Several severe cases occured. In areas of unstable transmission, pregnant women are more susceptible to severe falciparum malaria than their non-pregnant peers [16] and different manifestations of severe falciparum malaria including cerebral malaria have been reported among pregnant women of central Sudan [17].

The study showed no significant association between malaria and parity, which is also reported from other areas and locations with intense malaria transmission [10,12,18]. In areas where transmission is high and the level of acquired pregnancy immunity against malaria is expected to be significant, primigravidae will be more affected [1,2,11].

In contrast to the previous observations [10-12], age was not significantly associated with malaria in the present study. Lander *et al *have also reported no significant association between malaria infection and maternal age [18].

Women who attended the antenatal clinic in the third trimester had about a 1.5-fold higher risk of malaria parasitaemia. This is in line with some observations, although several studies report the second and early third trimesters as the time of peak prevalence [1,3,4,10-12]. Dicko *et al *reported the first trimester as the main risk period [19].

The mean haemoglobin was significantly lower in women with malaria infection and significantly lower in severe cases. Reduction of haemoglobin has been reported in areas of unstable transmission in Thailand and in Ethiopia, as well as in areas with stable transmission [3,6]. Regardless of transmission level and pre-pregnancy level of malaria immunity, maternal anaemia remains the most frequent consequence of malaria during pregnancy [4].

## Conclusion

The results suggest that prevalence of *P. falciparum *malaria is considerable in pregnant women in this part of Sudan and severe cases do occur. Preventive measures (chemoprophylaxis and insecticide-treated bednets) may be beneficial in this area for all women irrespective of their age or parity.

## Authors' contributions

IA carried out the study and participated in the statistical analysis and procedures, AHK participated in the statistical analysis, MIE coordinated and participated in the design of the study, statistical analysis and the drafting of the manuscript. All the authors read and approved the final version.
